# Prevalence, Cardiometabolic Comorbidities and Reporting of Chronic Kidney Disease; A Hungarian Cohort Analysis

**DOI:** 10.3389/ijph.2023.1605635

**Published:** 2023-03-30

**Authors:** Antal Zemplényi, Eszter Sághy, Anna Kónyi, Lilla Szabó, István Wittmann, Boglárka Laczy

**Affiliations:** ^1^ Center for Health Technology Assessment and Pharmacoeconomic Research, Faculty of Pharmacy, University of Pécs, Pécs, Hungary; ^2^ AstraZeneca Ltd., Budapest, Hungary; ^3^ Second Department of Medicine and Nephrology-Diabetes Center, University of Pécs Medical School, Pécs, Hungary

**Keywords:** comorbidities, prevalence of CKD, reporting of CKD, medical records, real-world data

## Abstract

**Objectives:** Chronic kidney disease (CKD) implies increased comorbidity burden, disability, and mortality, becoming a significant public health problem worldwide, however, prevalence data are lacking in Hungary.

**Methods:** We determined CKD prevalence, stage distribution, comorbidities using estimated glomerular filtration rate (eGFR), albuminuria, and international disease codes in a cohort of healthcare utilizing residents within the catchment area of the University of Pécs, in the County Baranya, Hungary, between 2011 and 2019 by database analysis. The number of laboratory-confirmed and diagnosis-coded CKD patients were compared.

**Results:** Of the total 296,781 subjects of the region, 31.3% had eGFR tests and 6.4% had albuminuria measurements, of whom we identified 13,596 CKD patients (14.0%) based on laboratory thresholds. Distribution by eGFR was presented (G3a: 70%, G3b: 22%, G4: 6%, G5: 2%). Amongst all CKD patients 70.2% had hypertension, 41.5% diabetes, 20.5% heart failure, 9.4% myocardial infarction, 10.5% stroke. Only 28.6% of laboratory-confirmed cases were diagnosis-coded for CKD in 2011–2019.

**Conclusion:** CKD prevalence was 14.0% in a Hungarian subpopulation of healthcare-utilizing subjects in 2011–2019, and substantial under-reporting of CKD was also found.

## Introduction

Chronic kidney disease (CKD) is increasingly acknowledged as an important public health problem. Recent reports cited the global CKD prevalence as 9.1% and 13.4%, corresponding to 700 million and one billion individuals worldwide (1, 2). CKD occurs in 15%–20% of the adult population, affecting 30%–40% of those over 70 years of age (1–5). The rising all-age prevalence of CKD is primarily driven by the increased prevalence of diabetes mellitus (DM), hypertension, obesity, and general aging of the world population (1–5). CKD is associated with adverse clinical outcomes and significantly reduced life expectancy. In addition to the high risk of progression to end-stage kidney disease (ESKD), CKD is recognized as an independent risk factor for cardiovascular (CV) and all-cause mortality (6–9).

CKD is defined as abnormalities of kidney function (below thresholds of estimated glomerular filtration rate, eGFR) or structure (e.g., abnormal albuminuria or imaging), present for more than 3 months, with implications for health (10, 11). CKD could be established without structural kidney impairment if the eGFR is lower than 60 mL/min/1.73 m^2^ (CKD stage G3 to G5) for at least 3 months (10, 11). The most recent KDIGO (Kidney Disease: Improving Global Outcomes) CKD guideline recommends the assessment of both eGFR and albuminuria for the diagnosis and classification of CKD stages (based on eGFR into six stages of G1, G2, G3a, G3b, G4, G5 and based on albuminuria into three stages of A1, A2, A3), denoting also the prognosis and risk stratification (G1<G5 and A1<A3) (10).

The relationship of lower eGFR and higher albuminuria with increased risk for CV disease (CVD), hospitalizations, heart failure, and mortality has been clearly demonstrated (6–9, 12–14). The excess mortality of advanced CKD that may exceed the incidence of ESKD (12, 15, 16), is predominantly caused by CVD, including coronary heart disease, stroke, and heart failure (6, 13, 14). Thus, as CKD progresses, the clinical and healthcare burden is attributable to the increasing presence of comorbidities and complications, although relatively minor proportions of patients belong to the high-risk and very high-risk CKD stages (2, 5, 10, 13, 17–20). The mapping of CKD epidemiology based on eGFR and albuminuria measurements could largely contribute to the evaluation of both ESKD and CVD risk of a studied population.

CKD in early stages is fairly asymptomatic. However, early detection and effective, evidence-based therapy of CKD could significantly improve both kidney and patient survival, and the quality of life of patients (13, 21). In recent years, considerable efforts have been made to increase the awareness of CKD, to timely detect and deliver proper care to identified CKD patients. CKD is still often under-detected, even amongst patients with high-risk conditions (e.g., DM, hypertension, or CVD) (22–25), where screening for CKD is recommended by regularly testing eGFR and albuminuria (10, 21, 26).

DM and hypertension are the dominant global etiologies of CKD, accounting for over 60% of ESKD cases (2) and are also closely associated with any CKD (3–5, 9, 10, 13, 17–19, 21, 22, 27–29). In a recent Swedish cohort study, the weighted prevalence of CKD (stages 1–5) was 26.2% in DM patients, 28.7% in hypertensive patients, and 36.8% in patients with CVDs in 2015–2018, showing decreasing proportions of patients by the CKD status with more severe stages for all comorbidities (19). About 40% of patients with DM (>90% type 2) have CKD, with higher rates (∼60%) in those over 65 years of age (5, 27, 28). By the USRDS data, overall prevalence of DM was 32.8% in CKD patients in 2015–2018 and was higher in stages G4-5 (46.8%) compared to stage G3 (32.3%); the prevalence of hypertension was 71.8% (5). Several subtypes of CVDs are 2–4 times as common in CKD patients compared to those without CKD (12, 13). The prevalence of CVDs was 75.3% in G4-5, 66.6% in G3, and 63.4% in G1-2 stages by the USRDS 2021 data (12). Of the CVDs, both eGFR and albuminuria were associated more robustly with heart failure and CV mortality than with coronary heart disease or stroke (13), consistent with higher costs related to renal events and heart failure than the costs for atherosclerotic events in CKD patients (18).

A growing body of studies have been reported on the CKD prevalence over the last few years, indicating also that estimates are largely affected by the CKD definition (e.g., based on single or repeated measurements of eGFR with or without albuminuria (for G1-5 or G3-5 stages), or by using diagnosis codes), and the population under study (e.g., screening in the general population, testing in targeted high-risk cohorts, or in cohorts under routine clinical care), among other factors (3, 5, 17–19, 30–33). For example, the CaReMe study across 11 countries in Europe, Canada and Israel identified 2.4 million CKD patients and showed that the pooled prevalence of possible CKD was 10.0% [8.7%–11.4%], based on single abnormal eGFR or albuminuria value or CKD diagnosis. The measured CKD was 7.0% [5.6%–8.5%], based on dual eGFR and albuminuria values by the KDIGO, while the prevalence of diagnosis coded CKD was 3.5% [2.6%–4.8%] (18). In this study only five countries had data on both measured and diagnosed CKD, and among the KDIGO-confirmed CKD patients only 34% had been diagnosed with CKD (18).

Prevalence estimates of CKD, from any source of available data, either laboratory-based or diagnosis coded studies, are useful indicators of CKD epidemics (3, 4, 12, 17, 18, 30, 33), by which optimal preventive and treatment strategies could be implemented to achieve reduced morbidity and mortality of CKD at the population level. Unfortunately, the public health efforts to survey CKD patients are hindered in Hungary, because there is no CKD registry, and to date, yet epidemiological analyses for CKD patients, not even at regional level, have not been conducted.

The objective of the present population-based CKD-EPI-HUN study was to determine the prevalence of CKD, the stage distribution and cardiometabolic comorbidities of CKD patients identified by the KDIGO definition, in a healthcare-utilizing cohort of residents having laboratory data in the catchment region of the University of Pécs, County Baranya, Hungary between 2011 and 2019. The numbers of laboratory-confirmed and diagnosis-coded CKD patients were compared to assess how well the diagnosis-coding captures the real population of laboratory-confirmed CKD patients.

## Methods

### Study Design and Data Collection

The CKD-EPI-HUN study was a retrospective, population-based epidemiological study using data collection. The study was conducted in the subpopulation of healthcare-utilizing residents living in Southern Hungary, in County Baranya within the catchment area of the University of Pécs, which is one of the largest healthcare providers in Hungary.

The University Medical Center provides tertiary care in the region, and secondary care in six districts of Baranya County (Komló, Pécs, Pécsvárad, Sellye, Siklós, Szentlőrinc). Residents of these districts are referred by their general practitioner for specialist care to the University, where their outpatient and inpatient clinical care is almost exclusively provided. Additionally, there is no other autonomous facility for laboratory testing in this area.

We used the electronic medical record (EMR) health database of the University of Pécs. Since 2008, the EMR system has been used to collect patient-level healthcare data, aggregated for about 1 million patients. The database contains information on demographics, diagnoses, laboratory tests, vital signs and prescriptions, each linked to a unique, anonymized patient identifier.

The study protocol was reviewed and approved by the Ethics Committee of the Faculty of Medicine, University of Pécs in November 2021 (reception number 9110-PTE 2022). Participants were not required to give written informed consent, as pre-existing anonymous data were collected from the University of Pécs Health Database.

### Study Population

**FIGURE 1:**
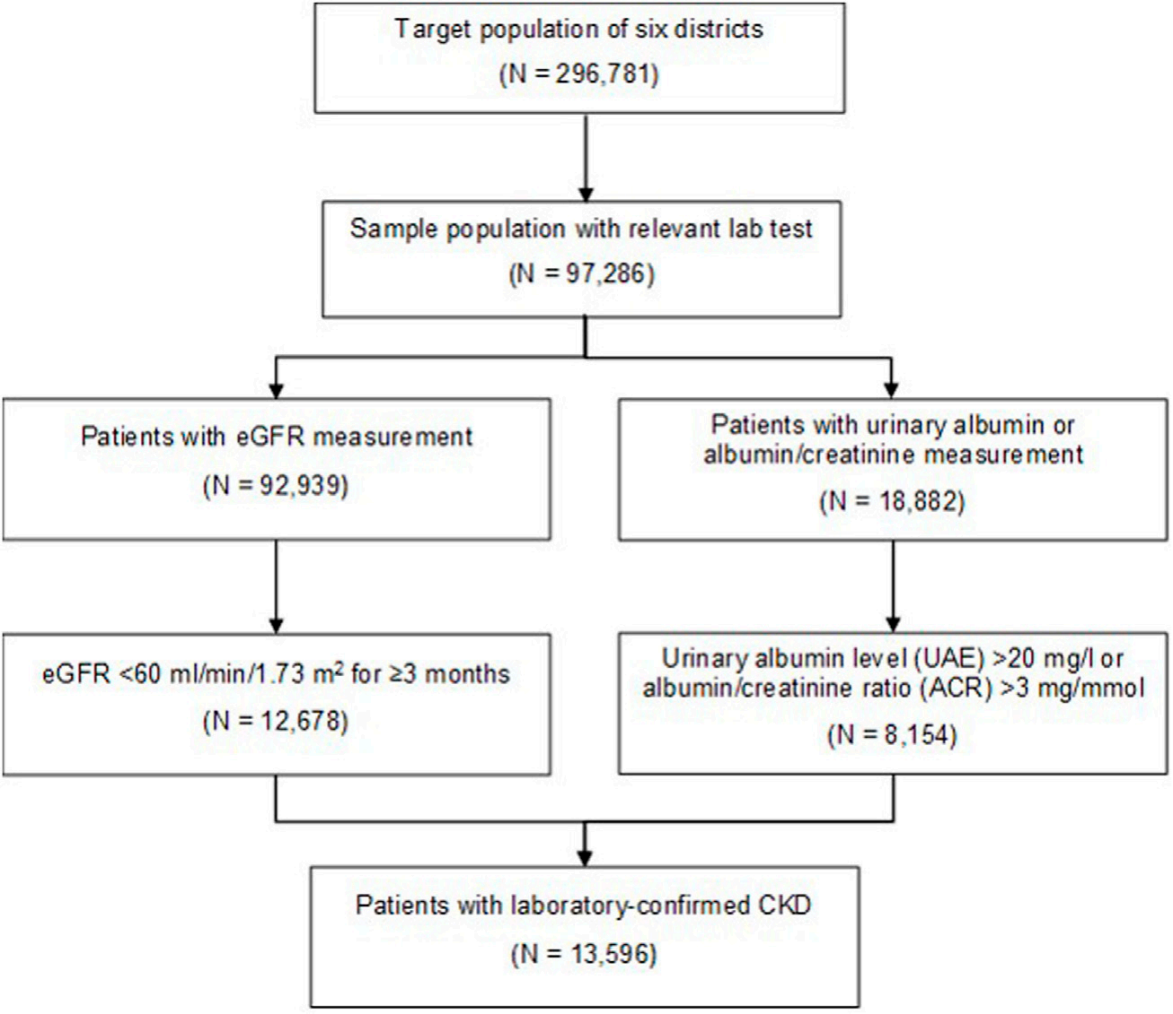
Flow diagram of patients with laboratory-confirmed chronic kidney disease (Prevalence, cardiometabolic comorbidities and reporting of chronic kidney disease, Hungary, 2011–2019).

Target population of the analysis included all subjects living in one of the six districts within the University of Pécs healthcare territory in 2019. Target population was under routine clinical practice. Total population of the six districts comprised of 296,781 people according to the 2018 Central Statistical Office register (34) (Figure 1), and could be considered representative of the Hungarian general population.

Among these residents, who were still alive and lived in one of the six districts in 2019, we examined those who had relevant laboratory measurements for CKD (eGFR and/or albuminuria testing) between 2011 and 2019 (called sample population, N = 97,286) (Figure 1).

CKD patients were subsequently identified based on their eGFR values, and urinary albumin excretion (UAE, mg/L) or albumin-to-creatinine ratio (ACR, mg/mmol) test results of single spot urine samples within the University database between 1st of January 2011 and 31st of December 2019. We assumed that if laboratory testing confirmed CKD during this period, it was still present in 2019. The prevalence of CKD was thus examined in 2019 (35). The number of laboratory-confirmed CKD patients was 13,596 (Figure 1).

By the KDIGO definition (10), patients were considered to have CKD when they have at least two measurements of eGFR below 60 mL/min at least 90 days apart (and without any measurements above 60 mL/min during this period). For patients who did not meet the eGFR criteria or, did not have eGFR measurement, CKD was determined based on two consecutive measurements for UAE over 20 mg/L, or ACR above 3 mg/mmol (10). eGFR, which is automatically coupled whenever serum creatinine is measured, was given by the CKD-EPI (CKD Epidemiology Collaboration) equation as recommended (10, 11).

## Data Analysis

### Prevalence Data

Crude prevalence data by age group and sex of the target district’s population were compared to our sample population (Table 1). As data by age group and sex of the district’s population could only be obtained for 2018 from the Central Statistical Office (34), and there was no significant change in the population of the districts in 2019 (35), we used the 2018 data in our analyses, and also for estimating the age and sex standardized CKD.

**TABLE 1:** Comparison of sample population and target population of the six districts stratified by sex and age (Prevalence, cardiometabolic comorbidities and reporting of chronic kidney disease, Hungary, 2011–2019).

Age group	Sample population (2019) (N = 97,286)		Sex distribution (2019) (%)		Target population of the six districts (2018) (N = 296,781)		Sex distribution (2018) (%)	
	Male	Female	Male	Female	Male	Female	Male	Female
0–19	724	748	0.7	0.8	27,936	26,353	9.4	8.9
20–29	2,366	3,290	2.4	3.4	18,262	18,119	6.2	6.1
30–39	3,658	5,267	3.8	5.4	20,088	19,477	6.8	6.6
40–49	6,633	8,296	6.8	8.5	23,847	23,413	8.0	7.9
50–59	7,550	8,955	7.8	9.2	19,184	19,999	6.5	6.7
60–64	5,169	6,089	5.3	6.3	10,449	12,105	3.5	4.1
65+	15,224	23,317	15.6	24.0	21,908	35,641	7.4	12.0
Total	41,324	55,962	42.5%	57.5%	141,674	155,107	47.7%	52.3%

The proportion of patients with laboratory-confirmed CKD was assessed in relation to the sample population (as measured CKD prevalence), and also the target district’s population standardized by age and sex (as estimated CKD prevalence) (Table 2).

**TABLE 2:** Prevalence of CKD in the target population of six districts standardized by sex and age (Prevalence, cardiometabolic comorbidities and reporting of chronic kidney disease, Hungary, 2011–2019).

Age group	Patients with CKD (N = 13,596)		Measured prevalence of CKD relative to the sample population (N = 97,286) (%)		Estimated prevalence of CKD in the six districts standardized by age and sex (N = 296,781)	
	Male	Female	Male	Female	Male	Female
0–19	108	110	14.9%	14.7%	4,167	3,875
20–29	115	123	4.9%	3.7%	888	677
30–39	113	135	3.1%	2.6%	621	499
40–49	317	254	4.8%	3.1%	1,140	717
50–59	657	557	8.7%	6.2%	1,669	1,244
60–64	628	678	12.1%	11.1%	1,269	1,348
65+	3,483	6,318	22.9%	27.1%	5,012	9,657
Total	5,421	8,175	13.1%	14.6%	14,766	18,018
	13,596		**14.0%**		32,784 (**11.05%**)	

### CKD Stages

We examined the distribution of identified CKD patients in the different stages of CKD at the time of the index date, defined as the date on which the patient was first diagnosed with CKD based on chronicity (Table 3). According to the KDIGO (10), CKD stages based on the eGFR were: G3a (eGFR: 45–59 mL/min/1.73 m^2^) mildly to moderately decreased; G3b (eGFR: 30–44 mL/min/1.73 m^2^) moderately to severely decreased; G4 (eGFR: 15–29 mL/min/1.73 m^2^) severely decreased; and G5 (eGFR <15 mL/min/1.73 m^2^) kidney failure. CKD stages based on albuminuria were: A1 (UAE: <20 mg/L or ACR: <3 mg/mmol) normal to mildly increased; A2 (UAE: 20–200 mg/L or ACR: 3–30 mg/mmol) moderately increased; and A3 (UAE: >200 mg/L or ACR: >30 mg/mmol) severely increased (10).

**TABLE 3:** Distribution of comorbidities in CKD stages by eGFR and in all laboratory-confirmed CKD patients (Prevalence, cardiometabolic comorbidities and reporting of chronic kidney disease, Hungary, 2011–2019).

Comorbidity	CKD stages by eGFR (N)				Comorbidities in stagesG3-G5 CKD patients		Comorbidities in all CKD patients	
	G3a	G3b	G4	G5	N	%	N	%
Diabetes mellitus (E10-E14)	2,400	887	279	103	3,669	39.1	5,643	41.5%
Hypertension (I10-15)	4,722	1,693	504	217	7,136	76.1	9,548	70.2%
Heart failure (I50)	1,376	639	207	76	2,298	24.5	2,781	20.5%
Myocardial infarction (I21-24)	625	274	99	34	1,032	11.0	1,283	9.4%
Stroke (I63-64)	800	295	69	17	1,181	12.6	1,426	10.5%
Total	9,923	3,788	1,158	447	9,377	100.0	13,596	100.0%

### Comorbidities

Frequent comorbidities, such as DM (E10-14), hypertension (I10-15), heart failure (I50), myocardial infarction (I21-24), and stroke (I63-64), were studied by having relevant ICD-10 (International Classification of Diseases, 10th version) diagnosis codes at least twice in the patient’s medical history, at least 90 days apart and within 1 year. The number of patients with these comorbidities were examined for the group of all identified CKD patients, and also separately in the group of eGFR-based CKD patients with distributions across G3 to G5 stages (Table 3).

We also measured the correlation between comorbidity and the severity of CKD using Kendall’s tau non-parametric rank correlation test, which is used to measure association between two ordinal variables. We calculated the sum of the five comorbidities (diabetes, hypertension, heart failure, myocardial infarction, and stroke) for each patient, resulting in a comorbidity score of 0 (none of the five diseases) to 5 (all five diseases). We categorized the CKD stages as 0 (normal), 1 (A2,G3a), 2 (G3b), 3 (A3,G4), and 4 (G5).

### CKD Reporting

In Hungary, ICD-10 code N18 and N19 are consistently used for reporting patients with CKD for administrative health insurance claims purposes. However, these codes are not always recorded for patients with laboratory-confirmed CKD.

To investigate the extent to which diagnosis codes are used to estimate the true number of patients with CKD, we analyzed how the number of patients with laboratory-confirmed CKD compares with the number of patients reported and diagnosis-coded for chronic kidney impairment (Table 4), including the diagnosis codes for chronic renal failure (ICD-10: N18) and un-specified renal failure (ICD-10: N19), which typically describes the disease in the study population.

**TABLE 4:** Accuracy of N18 and N19 coding to represent laboratory-confirmed CKD (Prevalence, cardiometabolic comorbidities and reporting of chronic kidney disease, Hungary, 2011–2019).

	N18, N19+	N18, N19−	Total		
CKD confirmed by laboratory test**+**	3,893	9,703	13,596	Sensitivity	28.6%
CKD not confirmed by laboratory test−	2,374	81,316	83,690	Specificity	97.2%
Total	6,267	91,019	97,286		

Data queries were performed using Microsoft SQL. All calculations and data generation for tables and graphs were performed using R version 1.4.1717 and Microsoft Excel.

## Results

### Subjects With CKD

Target population of the six study districts (Pécs, Pécsvárad, Komló, Sellye, Siklós, Szentlőrinc) totaled 296,781 persons, of which 31.3% had eGFR test (N = 92,939) and 6.4% had UAE or ACR test (N = 18,882). The sample population totaled 97,286 patients who had relevant eGFR and urinary albumin laboratory tests (Figure 1). The total number of patients who met the criteria for CKD was 13,596, representing 14.0% of the sample population as measured CKD prevalence (Figure 1). Characteristics of the sample population are presented in Table 5.

**TABLE 5:** Characteristics of patients of the sample population with relevant laboratory data (Prevalence, cardiometabolic comorbidities and reporting of chronic kidney disease, Hungary, 2011–2019).

Category	Parameter	Sample population (N = 97,286)	(%)
Sex	Male	41,324	(42.5)
	Female	55,962	(57.5)
Age Categories	0–19	1,472	(1.5)
	20–29	5,656	(5.8)
	30–39	8,925	(9.2)
	40–49	14,929	(15.3)
	50–59	16,505	**(**17.0)
	60–64	11,258	(11.6)
	65+	38,541	(39.6)
Districts	Komló	15,759	(16.2)
	Pécs	52,300	(53.8)
	Pécsvárad	53,13	(5.5)
	Sellye	6,611	(6.8)
	Siklós	11,145	(11.5)
	Szentlőrinc	6,158	(6.3)
CKD	Laboratory-confirmed CKD	13,596	(14.0)
	No laboratory-confirmed CKD	83,690	(86.0)
CKD stages	A2 Moderately increased albuminuria	3,962	(4.1)
	A3 Severely increased albuminuria	257	(0.3)
	G3a Midly to moderately decreased eGFR	6,569	(6.8)
	G3b Moderately to severely decreased eGFR	2,024	(2.1)
	G4 Severely decreased eGFR	552	(0.6)
	G5 Kidney failure	232	(0.2)
	No laboratory-confirmed CKD	83,690	(86.0)
CKD with comorbidities	Diabetes mellitus (E10-14)	16,792	(17.3)
	Hypertension (I10-15)	37,603	(38.7)
	Heart failure (I50)	7,333	(7.5)
	Myocardial infarction (I21-24)	3,433	(3.5)
	Stroke (I63-64)	5,205	(5.4)
CKD diagnosis codes	N18: Chronic renal failure	3,226	(3.3)
	N19: Unspecified renal failure	3,041	(3.1)
	No CKD-relevant diagnosis code recorded	91,019	(93.6)

The sample included patients who had used the University Center controlled medical services for any health problem or other tests, and therefore differed slightly from the total population of the six districts. The sample population has a higher proportion of women (57.5% vs. 52.3%) and a higher proportion of middle-aged and older population (over 65 years 39.6% vs. 19.4%). Table 1 shows a comparison of the two populations stratified by age and sex.

As CKD was more prevalent in women and older age groups, the prevalence of CKD in the six districts was estimated by standardizing the data by age and sex, as shown in Table 2. The estimated standardized prevalence of laboratory-confirmed CKD in the target population was 11.0% (Table 2).

### CKD Stages

The proportions of identified CKD patients based on their eGFR levels (N = 9,377) were the following in the CKD stages: 70% in stage G3a, 22% in stage G3b, 6% in stage G4 and only 2% in stage G5 (Table 5). The number of dialyzed patients amongst G5 patients was 61. The average age of CKD patients identified by eGFR was 75 years, of which 34.5% were males (data not shown).

The distribution of CKD patients based on their UAE and ACR level (N = 4,219) showed that 94% were in stage A2 and 6% in stage A3 (Table 5). The average age of CKD patients identified by UAE and ACR was 58 years, and the proportion of males was 52% (data not shown).

### Comorbidities

We assessed the comorbidities of patients for the entire group of CKD patients identified by both eGFR and albuminuria (N = 13,596), of which 41.5% had known diabetes, 70.2% had hypertension, 20.5% had heart failure, 9.4% had myocardial infarction, and 10.5% had stroke (Table 3). The distribution of examined comorbidities in stages G3 to G5 for the eGFR-based group of CKD patients is shown in Table 3.

The association between comorbidity and CKD stages were highly significant (*p*=<0.001) with a Kendall’s tau value of 0.1517, which indicates moderate monotonicity between the two variables. This indicates that the more comorbidities a patient has (out of the five studied diseases), the more severe the patient’s kidney impairment (data not shown).

### CKD Reporting

During the study period (2011–2019), we found that there is a substantial under-reporting of CKD, as only 28.6% of laboratory-positive cases (N = 13,596) were coded with N18 or N19 (N = 3,893), taking into account all diagnosis types, including principal, secondary, or prescription diagnoses (Table 4).

In addition to the patients identified with diagnosis codes of N18 or N19 (N = 6,267), there were 9,703 patients who would have been classified to have CKD based on their laboratory results of eGFR and albuminuria only, adding up to 15,970, which is 16.4% of the sample population (Table 4). In this case, the estimated age and sex standardized CKD prevalence is 12.5% in the target population of the six districts.

## Discussion

This study is the first to report prevalence data of CKD in a Hungarian subpopulation, which—based on laboratory results—was 14.0% (impacting 13,596 subjects) in the studied population and 11.0% standardized by age and sex. A total prevalence of CKD, including the number of all laboratory-positive and ICD diagnosis-coded patients (N = 15,970) could be estimated 16.4% in the studied population and 12.5% standardized by age and sex. We also found that 28.6% of laboratory-confirmed CKD patients were only diagnosis-coded, showing under-reporting of CKD in our studied population.

A wide variation in the standardized prevalence rates of CKD was reported in different populations across the European countries, ranging from 3.3% in Norway to 17.3% in northern Germany (32), but Hungary has not been represented so far. In a recent meta-analysis of 100 studies (2), the global mean CKD prevalence was estimated 13.4% for G1-5 stages and 10.6% for G3-5 stages. For Europe, the mean prevalence of CKD was estimated 18.4% for G1-5 stages and 11.9% for G3-5 stages (2). As to date, there were no available data in Hungary. According to the latest estimates for Hungary by the Global Disease Burden study (1), the number of CKD patients was 1,323,316 (95% uncertainty interval: 1,226,092 and 1,433,403) in 2017, which may have further increased between 2017 and 2019 given the 1.1% growth rate of CKD incidence (1). Thus the crude prevalence of CKD in Hungary could be estimated ∼13.8%, based on the total national population (N = 9,772,756) in 2019 by the Central Statistical Office (35). Our findings show that standardized prevalence data (lab-based 11.0%, ICD+lab-based 12.5%) were lower than the European mean prevalence or the latest international estimates for Hungary, which appeared more comparable with our results of the studied sample population (lab-based 14.0%, ICD+lab-based 16.4%).

In this retrospective report only patients who had laboratory tests at the University of Pécs were analyzed, which covers ∼33% of the population of the geographical area studied, and the target population was under routine clinical practice, thus specific reasons for testing could not be ascertained. Therefore, there is a possibility for the overrepresentation of specific subsets of patients, who more frequently used medical services, and were more likely to be tested (e.g., those at high-risk for CKD with DM, heart failure, and elderly). Hence the proportion of CKD among these patients cannot be extrapolated to those who did not have any test results. On the other hand, true prevalence of CKD in the region might be even higher if we take into account the missing CKD cases amongst individuals with no kidney measures. The probability weighting against untested subjects, accounting for nonrandom testing for eGFR and albuminuria, has been shown to decrease the prevalence of CKD (from 18.6% to 10.6%) within the Stockholm area population (19).

Few studies have reported nationwide prevalence data using the KDIGO criteria (5, 17, 18, 36–39). In the US adult population, the prevalence of CKD has been relatively stable at 14.4% over the last several years, based on a single abnormal eGFR or albuminuria test (5). From other non-European countries, CKD prevalence in adults was 10.8% in China, and 11.9% in Taiwan (38, 39). By recent studies from Europe, CKD prevalence was 15.1% in Spain, based on a single eGFR or albuminuria test (36), but it was only 6% in Iceland where chronicity was taken into account (37). In the CaReMe study (18), the KDIGO-confirmed prevalence of CKD was available for five countries (with a pooled prevalence of 7.0%), including Belgium (5.6%), Canada (7.0%), Israel (6.5%), Portugal (9.8%), and Sweden (6.1%). In all of these countries the albuminuria-confirmed CKD cases were inferior to the eGFR-confirmed cases (2.4% vs. 4.6%) (18). We also confirmed persistence by dual measurements of eGFR (CKD-EPI) and albuminuria (spot urine ACR or UAE) at least 3 months apart, which could underestimate the real prevalence.

Using the KDIGO definition, by assessing both eGFR and albuminuria, enabled us to examine the stage distribution of CKD patients, although cross-classifying could not have been performed due to the low number of cases. The majority of patients had normal and mildly decreased kidney function (G1-2 stages, N = 80,261, 82.5% of studied population) and had normal albuminuria (A1 stage, N = 10,728, 11% of studied population), in agreement with previous population-based studies (5, 8, 17–19, 36, 38). The distribution of CKD patients identified by eGFR (G3a: 70%, G3b: 22%, G4: 6%, G5: 2%) showed the largest proportion in stage G3, and decreasing proportions in more severe stages (G3>G4>G5), broadly consistent with other reports from both general and at risk population studies (2, 5, 8, 15, 17–19, 22, 36, 40, 41). The distribution of CKD patients based on abnormal UAE and ACR, showed a relatively higher proportion of patients in stage A2 (94%) than stage A3 (6%), which may be due to an increased number of tested individuals who suffer from such underlying diseases that are often accompanied with moderately increased albuminuria.

We provided prevalence estimates for CKD-associated key comorbidities. Amongst all identified CKD patients, 41.5% had known diabetes, 70.2% had hypertension, 20.5% had heart failure, 9.4% had myocardial infarction, and 10.5% had stroke. However, ICD-based evaluation of comorbidities tend to underestimate the real prevalence. Our data seem similar to the CaReMe study results of more than one million KDIGO-confirmed CKD patients, of whom 38.0% had DM, 15.8% had heart failure, 21.4% coronary artery disease, and 11.8% had stroke (18). The absolute number of affected patients decreased by the CKD status (G3a>G3b>G4>5) in case of all examined comorbidities. Although, we found that severity of CKD significantly correlated with the co-presence of more comorbidities.

The proportion of DM patients in this study (G3-5: 39.1% and G1-5: 41.5%) well-approximated the prevalence data known for CKD-associated DM in the literature (5, 27, 28). The highest proportion of CKD patients had hypertension (G3-5: 76.1% and G1-5: 70.2%), which is also consistent with literature data (5, 15, 42). Given the older age of identified CKD patients in this study and that ICD-based comorbidities were examined, our findings are somewhat close to the report of the 2019 US Medicare FFS administrative data showing that the overall prevalence of DM was 50.0%, hypertension 91.9%, heart failure 24.9%, myocardial infarction 9.2%, cerebrovascular disease 15.6% in older (aged ≥66 years) patients (12).

In the CaReME study, 34% of laboratory confirmed CKD cases were diagnostically coded (18). The CKD prevalence among older US patients was about 2.5-fold higher based on NHANES laboratory data (∼37%) than it was based on diagnosis codes (∼14%) from administrative Medicare claims (12). The validity testing of the Dutch Vektis database showed 27% sensitivity to capture CKD patients with eGFR below 60 mL/min/1.73 m^2^, while the positive predictive value was 90% (33). Here we also analyzed how the number of laboratory-confirmed CKD patients compares with the number of ICD-coded patients. We found that ICD codes covered only 28.6% of those patients whose CKD was confirmed by laboratory results during the study period (2011–2019), and the actual number of CKD patients were ∼2-fold higher than what was reported from ICD codes (14% vs. 6.4% of the study population). Our data are very similar (sensitivity: 28.6%, specificity: 97.2%) to other reports, indicating substantial under-reporting of patients with CKD.

The under-coding of CKD was consistently observed in most countries (18, 31, 33, 37, 39), contributing to the lower awareness of CKD. In general, lower awareness of CKD may be a result of failure to screen by measuring eGFR and albuminuria, to recognize CKD patients if tested, and to document diagnosis if identified, or any combination of these. CKD is asymptomatic and highly prevalent in early stages, thus screening and management of early stages patients usually takes place in primary-care practices. In this study, about one-third of the six district’s population had relevant eGFR, UAE or ACR laboratory measurements. The current guideline indorses the screening of CKD in patients with well-accepted risk factors, such as hypertension, DM, CVDs, obesity, positive family history, and older age (10, 21), instead of population-wide screening programs. We found a higher rate of eGFR testing (31.3%) than albuminuria testing (6.4%) in this study, suggesting that albuminuria is less commonly measured than the kidney function in the routine clinical practice.

Yet, several studies from primary-care practices showed the underutilization of tests, inadequate recognition, and under-coding of CKD patients, even in those with high-risk conditions (16, 23–25, 42–44). In the CURE-CKD registry, nearly one half of patients with persistently low eGFR remained undiagnosed in 2014–2017 (22). In contrast, higher rates of testing for serum glucose and lipids, and higher sensitivity of coding for DM were established, suggesting that clinicians are less aware of CKD or its relationship to CVD than that of other CVD risk factors (44). Here we found that only 28.6% of lab-confirmed CKD cases were reported by ICD codes in 2011–2019. Together these data support the unfortunate fact that substantial proportion of advanced CKD patients (∼30%–50%) enter the dialysis without prior referral and proper nephrology care (12). ICD coding of CKD should be encouraged if patients meet CKD criteria, so that CKD surveillance could be improved.

The strengths of this study are the representativeness of subjects at regional level with real-world data, standardization of data of the studied population, the large sample size including institutionalized patients, the use of international standard methods with confirmed persistency of CKD by the KDIGO definition, and the comparison of laboratory-based and ICD-based number of CKD cases.

This study has also some limitations. Only patients who had laboratory tests at the University of Pécs were analyzed, which covers about 33% of the population of the geographical area studied. We cannot exclude that some specific subgroups of patients were overrepresented in the study population. Standardized data involved basic demographic data of age and sex, other factors or diseases (e.g., comorbidities) of the target population were not evaluated. We did not study patients who had only one measurement below 60 mL/min/1.73 m^2^ but could potentially have CKD, thus there may be additional patients with CKD who did not meet the CKD criteria due to missing tests. In this retrospective database study, clinical data, specific reasons for testing, CKD etiology, other determinants of CKD (e.g., urine sediment, histology, imaging), drug therapy, or CKD progression (stage was established based on the first fulfilled criteria) were not determined.

In conclusion, this CKD-EPI-HUN study is the first to provide prevalence data of CKD in a Hungarian subpopulation using the KDIGO definition. The measured CKD prevalence was 14.0% among patients having medical records within a well-defined territory of the University laboratories, which—after standardization by age and sex, was estimated 11.0% in the study population. A total prevalence of CKD, including the number of all laboratory-confirmed and ICD-coded patients could be estimated 16.4% and 12.5% standardized by age and sex. These data suggest a relatively higher prevalence of CKD in Hungary. Our findings may be valuable for clinicians, policymakers, and entities focused on public healthcare to ensure a comprehensive approach to patient management in CKD.
